# Dissociated Spatial-Arithmetic Associations in Horizontal and Vertical Dimensions

**DOI:** 10.3389/fpsyg.2017.01741

**Published:** 2017-10-04

**Authors:** Dixiu Liu, Tom Verguts, Mengjin Li, Zekai Ling, Qi Chen

**Affiliations:** ^1^School of Psychology, South China Normal University, Guangzhou, China; ^2^Center for Studies of Psychological Application, South China Normal University, Guangzhou, China; ^3^Guangdong Key Laboratory of Mental Health and Cognitive Science, South China Normal University, Guangzhou, China; ^4^Department of Experimental Psychology, Ghent University, Ghent, Belgium

**Keywords:** numerical cognition, arithmetic, spatial attention, horizontal, vertical

## Abstract

Spatial–numerical associations (small numbers—left/lower space and large numbers—right/upper space) are regularly found in elementary number processing. Recently, the interest in this phenomenon has been extended from elementary number processing to mental arithmetic. Many studies have demonstrated horizontal spatial-arithmetic associations, i.e., solving addition or subtraction problems cause spatial shifts of attention rightward or leftward, respectively. However, the role of this effect in the vertical dimension has not been addressed. This is problematic because it leaves the analogy between elementary number processing and arithmetic incomplete. In order to make a strong case for a similarity between elementary number processing and mental arithmetic, a spatial-arithmetic association should be observed in the vertical dimension too. Here, we adopted the target detection paradigm from Liu et al. (2017) to replicate the horizontal spatial-arithmetic association, and meanwhile investigate whether this effect also exists in the vertical direction. Our results confirmed that addition could induce covert movement to right side and subtraction to left side. However, such a spatial-arithmetic association was not found in the vertical dimension. The implication of these findings is discussed.

## Introduction

In the past two decades, research has revealed a close link between number and space in elementary number processing (e.g., Dehaene et al., 1993; Fischer et al., 2003; Hubbard et al., 2005; Wood et al., 2008; Chen and Verguts, 2010). For example, in the seminal study of Dehaene et al. (1993), it was demonstrated that (in parity judgment) small numbers are associated with faster left-hand response and larger numbers with faster right-hand responses. This suggested a spatial-numerical association in horizontal space.

Spatial-numerical associations also exist in vertical space. In particular, there is a preference to associate small numbers with the lower part of space and larger numbers with the upper part (Schwarz and Keus, 2004; Gevers et al., 2006; Winter et al., 2015). For example, using eye movements, Schwarz and Keus (2004) found that responses to the lower space start earlier with smaller than with larger numbers; whereas responses to the upper space start earlier with larger than with smaller numbers. Evidence for a vertical spatial–numerical association also comes from the random number generation task, in which small numbers generation is associated with downward movement and large numbers generation with upward movement (Schwarz and Keus, 2004; Loetscher et al., 2010; Hartmann and Mast, 2012; Winter and Matlock, 2013). These studies suggest that there is a link between numbers and spatial bias in the vertical dimension. Hence, basic single-number processing has a well-established association with both the horizontal and the vertical dimension.

Recently, the focus of spatial–numerical associations has been extended from elementary number processing to mental arithmetic (McCrink et al., 2007; Knops et al., 2009b; Chen and Verguts, 2012; Fischer and Shaki, 2014; Masson and Pesenti, 2014). It has been reported that mental arithmetic causes spatial response biases (Knops et al., 2009a,b). Specifically, subjects prefer to select options on the right side after solving addition problems, and options on the left side after solving subtraction problems (Knops et al., 2009b; Marghetis et al., 2014). Moreover, performing addition or subtraction causes spatial shifts of attention rightward or leftward, respectively (Dormal et al., 2014; Masson and Pesenti, 2014; Mathieu et al., 2016; Liu et al., 2017; Masson et al., 2017). Using a target detection paradigm, Masson and Pesenti (2014) found that solving subtraction problems facilitates the detection of left-side targets (Experiment 1), whereas addition facilitates the detection of right-side targets (Experiment 2). This suggests that there is a close relationship between arithmetic problem solving and visuospatial attention orientation (Masson and Pesenti, 2014). These spatial shifts of covert attention in mental arithmetic are further confirmed and shown robustly at 300 ms after the arithmetic operation (Mathieu et al., 2016; Liu et al., 2017).

Such findings suggest a strong similarity between elementary number processing (e.g., Fischer et al., 2003) and mental arithmetic, and possibly similar cognitive mechanisms across these diverse tasks. However, to make this argument compelling, it needs to be demonstrated that a vertical (upper/lower) association also exists in mental arithmetic. Unfortunately, however, the investigation of spatial-arithmetic associations in the vertical dimension remains scarce. By analyzing spontaneous eye movements, it was found that during addition problems, the gaze is more directed upward than during subtraction (Hartmann et al., 2015; Masson et al., 2017). Furthermore, several studies indicate that vertical motions facilitate calculation in a congruent condition (i.e., solving addition problems when performing an upward motion or solving subtraction problems when performing a downward motion), but interfere with calculations in an incongruent condition (i.e., solving addition problems when performing a downward motion, or solving subtraction problems when performing an upward motion) (Lugli et al., 2013; Wiemers et al., 2014). These motion-arithmetic compatibility effects suggest that spatial-arithmetic associations may also occur in a vertical dimension.

**FIGURE 1:**
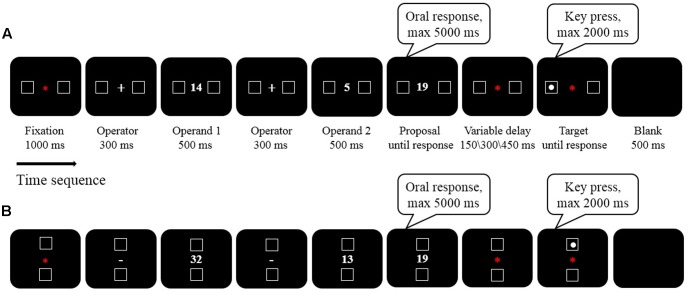
Task sequence and timing of a sample trial. The operator, first operand, second operand and the proposed result were sequentially presented at the center of the screen. The proposal would not disappear until participants give an oral response through a headset microphone, but with a maximum duration of 5000 ms. The target (a white solid circle) was randomly presented in one of the two boxes on 80% of all trials. Targets disappear as soon as participants respond, or remain on the screen with a maximum duration of 2000 ms. **(A)** horizontal dimension, the two boxes were bilaterally located on the left (or right) side of the fixation; **(B)** vertical dimension, the two boxes were flanked on the lower (or upper) side of the fixation.

If arithmetic operations are closely associated with space in vertical dimension, this raises the question of whether mental arithmetic can also induce a shift of attention to the upper or lower visual field. To address this question, in the present study we were the first to use symbolic magnitudes to investigate the spatial shifts of attention in both horizontal and vertical space in a within-subject design. This allowed us to investigate the strength of both horizontal and vertical associations, respectively, and compare them directly. If spatial-arithmetic associations have the same origins as those in single-number processing, the effect should at least be very robust in both dimensions. Thus, we adopted the target detection paradigm from our previous study (see Experiment 1 in Liu et al., 2017). A trial time course can be seen in **Figure 1**. Participants were instructed to perform two tasks: (1) solving an arithmetic problem and making a judgment on whether the proposed result (proposal) was correct or incorrect (mental arithmetic task); and (2) detecting whether the target (a white solid circle) was present or not (target detection task). The targets might appear horizontally (**Figure 1A**) or vertically (**Figure 1B**) arranged on bilateral sides of the fixation. RTs of detection response to targets were measured. In both horizontal and vertical conditions, the target detection task was preceded by a mental arithmetic task, and the magnitudes of the second operands and the proposals were matched for addition and subtraction. Furthermore, three variable delay (150, 300, and 450 ms) were introduced before target detection to investigate the time course in which spatial attention shifts were induced.

## Materials and Methods

### Participants

Based on the effect size of our previous study (Liu et al., 2017), we used G^∗^power^1^ to calculate the required sample size of this study in order to achieve a power of 95%. 27 undergraduates (13 male, 27 right-handed) took part in this experiment. They ranged in age from 19 to 24. All participants had normal or corrected-to-normal vision, and were naïve with respect to the objective of this study. These criteria for sample size and data quality are similar to our previous work (Liu et al., 2017).

The present study was approved by the Human Research Ethics Committee for Non-Clinical Faculties, School of Psychology, South China Normal University. The procedures and other relevant details of this experiment were in accordance with the approved guidelines as well as the ethical guidelines. We obtained informed consent from all subjects before the experiment.

### Materials and Design

The arithmetic problems used in the present study were identical to those in Liu et al. (2017, Experiment 1), which were constructed with the criteria used by Knops et al. (2009b). All stimuli appear in **Table 1**.

**Table 1:** All arithmetic problems presented and their correct and deviant results.

Operands		Proposals				
O1	O2	1/1.4	1/1.2	1/1	1.2/1	1.4/1
**Addition**
14	5	13	16	19	23	27
14	7	15	18	21	25	30
14	11	18	21	25	30	35
28	7	25	29	35	42	49
28	13	29	34	41	49	58
28	21	35	41	49	58	69
56	13	49	58	69	82	98
56	28	59	71	84	100	119
56	42	69	82	98	117	139
**Subtraction**
32	13	13	16	19	23	27
32	11	15	18	21	25	30
32	7	18	21	25	30	35
64	29	25	29	35	42	49
64	13	29	34	41	49	58
64	15	35	41	49	58	69
128	59	49	58	69	82	98
128	44	59	71	84	100	119
128	30	69	82	98	117	139

Stimuli were presented in a mixed design in which the dimension factor (horizontal or vertical) was blocked (see below); other factors, namely arithmetic operation (addition and subtraction), target side (left and right in the horizontal condition; lower and upper in the vertical condition), and delay (150, 300, and 450 ms) were randomly mixed within a block on a trial-to-trial basis. Each participant completed 720 trials. 20% of the trials were catch trials where no target appeared. The experiment was divided in two sessions. Each session contained four blocks (two horizontal and two vertical). Sessions were counterbalanced in an ABBA format. Half of the subjects did first the horizontal and then the vertical block in session 1; and then the vertical followed by the horizontal block in session 2. After a session finished, participants were asked to go outside the laboratory to have a compulsory rest of about 10 min so their eyes could rest. Accordingly, they were asked not to use their smart phone or other electronic device during the break. Between two blocks, a short rest was introduced. The experiment lasted about 80 min for each participant.

### Task and Procedure

The experiments were conducted on a Lenovo PC equipped with a 23-inch screen. Stimulus presentation and data collection were programmed by E-Prime 2.0 software.

The sequence and timing of a trial was similar to that in Liu et al. (2017); see **Figure 1**. A trial began with a red fixation dot “_∗_” (Calibri 20 pt, 4 mm, 0.4°) on the center of the screen for 1000 ms together with two flanking boxes. In the horizontal condition, the two boxes (each with 4.5° eccentricity, 1 cm × 1 cm, 1° × 1°) were at the left and right side of the fixation dot (**Figure 1A**), while in the vertical direction the two boxes (each with 4° eccentricity, 1 cm × 1 cm, 1° × 1°) were located above and below the fixation dot (**Figure 1B**). As soon as the fixation dot was removed, an operator sign (+ or -) (Calibri 40 pt, 8 mm, 0.8°) replaced the fixation dot for 300 ms to indicate the subsequent operation to be performed. Then, the first operand (O1, 500 ms), operator sign (Operator, 300 ms), second operand (O2, 500 ms) and a proposed result were successively presented (each digit was in Calibri 36 pt, 1.1 cm × 0.6 cm, 1° × 0.6°). Participants were instructed to make an oral judgment as quickly and as accurately as possible on whether the proposal was correct or incorrect [Dui (Yes) or Cuo (No)]. The proposal remained on screen until response; this was followed by a variable delay (150, 300, 450 ms), which was subsequently followed by the presentation of a target (white solid circle, 0.7° diameter) in one of the boxes (left or right box in the horizontal condition; lower or upper box in the vertical condition) on 80% of all trials. The other 20% of the trials were catch trials where no target appeared, so as to prevent anticipatory responses. Observers were asked to respond with their preferred hand on the space bar as quickly as possible once they had detected the target.

Before testing, participants were informed that arithmetic operations were irrelevant to target detection. They were required to keep their eyes fixed on the center of the screen during the task. There was a training session consisting of 24 practice trials before the first experimental block.

### Data Analysis

First, trials with error responses (to either arithmetic or target detection task) were discarded, and the following trials were also excluded from the analysis: (1) trials where the microphone failed to trigger or the judgment RT to the proposal was more than 5000 ms; and (2) trials where the RT was smaller or larger than three standard deviations from the mean for each participant. Second, two 2 × 2 × 3 repeated measures analysis of variance (ANOVA) were conducted to test the spatial-arithmetic association in different dimensions, with operation (addition, subtraction), target side (in the ANOVA for horizontal dimension: left, right; in the ANOVA for vertical dimension: lower, upper) and delay (150, 300, and 450 ms) as within-subject factors. Third, *post hoc* planned comparisons for each delay were subsequently conducted to test the interaction between arithmetic and target side, regardless of whether the operation × target side × delay interaction was significant or not. These analyses were used to identify the time course in which spatial-arithmetic association occurred.

Finally, in order to compare the spatial-arithmetic association effect across different dimensions, a 2 × 2 × 2 × 3 repeated measures ANOVA was carried out, with dimension (horizontal, vertical), operation (addition, subtraction), target side (congruent with operational momentum, incongruent with operational momentum), and delay (150, 300, and 450 ms) as within-subject factors.

Here we only report the target detection task data. See **Table 2** for a full list of the mean RT (and SD) for target detection across different dimensions. The mental arithmetic data can be seen in **Table 3**.

**Table 2:** Mean RT (and SD) of the target detection task as a function of Operation, Target side, and Delay (in ms) in horizontal and vertical dimension.

		Addition			Subtraction		
		150	300	450	150	300	450
**Horizontal**						
	Left	395 (65)	365 (72)	364 (67)	384 (50)	358 (60)	359 (51)
	Right	380 (64)	354 (65)	360 (64)	389 (60)	371 (61)	360 (59)
**Vertical**						
	Upper	396 (62)	366 (63)	376 (53)	403 (53)	364 (56)	372 (62)
	Lower	399 (60)	376 (62)	378 (55)	399 (57)	365 (59)	370 (53)

**Table 3:** Mean RT (and SD) of the proposal judgment task as a function of Operation, Target side, and Delay (in ms) in horizontal and vertical dimension.

		Addition			Subtraction		
		150	300	450	150	300	450
**Horizontal**			
	Left	707 (141)	713 (163)	699 (139)	798 (188)	787 (180)	825 (193)
	Right	708 (156)	706 (165)	723 (129)	826 (182)	838 (193)	851 (186)
**Vertical**			
	Up	745 (188)	716 (152)	752 (157)	859 (238)	835 (207)	861 (218)
	Down	715 (144)	726 (168)	731 (143)	800 (182)	813 (171)	819 (184)

## Results

### Horizontal Spatial-Arithmetic Association

**FIGURE 2:**
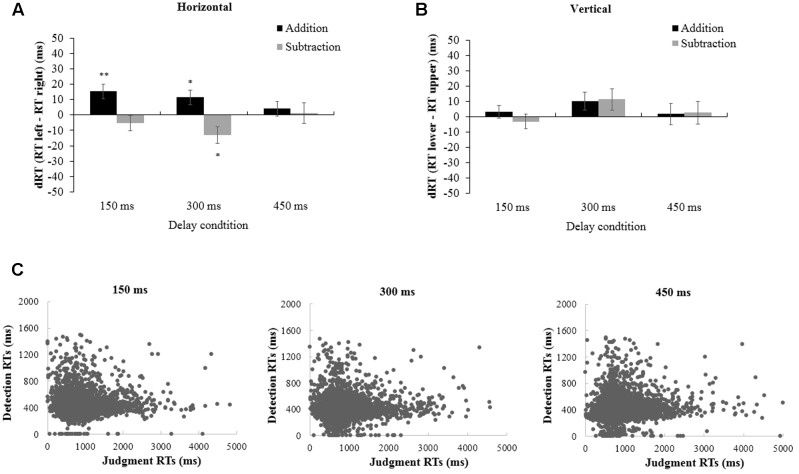
The difference in dRT as a function of operation (addition or subtraction) with 150, 300, and 450 ms delay in horizontal and vertical dimension. **(A)** dRT = RT (left)–RT (right), “up” means “right faster,” while “down” means “left faster”; **(B)** dRT = RT (lower)–RT (upper), “up” means “upper faster,” while “down” means “lower faster”; **(C)** All individual oral latencies (Judgment RTs) against target detection times (Detection RTs) in different delay conditions (150, 300, and 450 ms). Error bars represent standard error of the mean (SEM). ^∗^*p* < 0.05, ^∗∗^*p* < 0.01. Here and elsewhere, data for the target detection task only are shown (see main text).

The results appear in **Figure 2A**. There was a main effect of delay, *F*(2,52) = 26.343, *p* < 0.001, η^2^ = 0.503. Mean RTs were fastest in the 450 ms condition, which were significantly faster than in the 150 ms condition, *F*(1,26) = 31.958, *p* < 0.001, η^2^ = 0.551, but not different from the 300 ms, *F*(1,26) = 0.163, *p* = 0.690, η^2^ = 0.006. Importantly, we found a significant interaction between operation and target side, *F*(1,26) = 10.133, *p* < 0.01, η^2^ = 0.280. The simple effect analysis showed that after addition, right-side targets were detected faster than left-side targets, *F*(1,26) = 12.141, *p* < 0.01, η^2^ = 0.318. After subtraction, detection of left-side targets was faster than for right-side targets, but this facilitation effect was not significant, *F*(1,26) = 2.401, *p* = 0.133, η^2^ = 0.085. Other main effects or interactions were not found.

Follow-up planned comparisons revealed that in the 150 ms delay condition, addition facilitated the response to right-side targets relative to left-side targets, *F*(1,26) = 10.626, *p* < 0.01, η^2^ = 0.290. A tendency for a spatial bias to left-side targets was found after solving subtraction problems, but the difference of RTs to left and right side targets was marginally significant, *F*(1,26) = 1.078, *p* = 0.309, η^2^ = 0.040. For 300 ms delay, the spatial biases were found in both arithmetic operations, showing that right-side targets were detected faster after solving addition problems, *F*(1,26) = 5.809, *p* < 0.05, η^2^ = 0.183; while left-side targets were detected faster after solving subtraction problems, *F*(1,26) = 5.662, *p* < 0.05, η^2^ = 0.179. In 450 ms delay condition we did not find the interaction between arithmetic and target side (**Figure 2A**).

### Vertical Spatial-Arithmetic Association

The same statistical analyses were conducted to investigate the vertical spatial-arithmetic association (**Figure 2B**). In 2 × 2 × 3 repeated measures analysis of variance (ANOVA), the effect of delay was also significant, *F*(2,52) = 29.579, *p* < 0.001, η^2^ = 0.532, showing that in the 450 ms condition, the mean RT was significantly faster than that in 150 ms condition, *F*(1,26) = 24.569, *p* < 0.001, η^2^ = 0.486, but not different from the 300 ms, *F*(1,26) = 2.176, *p* = 0.152, η^2^ = 0.077. However, we did not find an interaction between operation and target side in the vertical dimension, *F*(1,26) = 1.315, *p* = 0.262, η^2^ = 0.048. Further comparisons disclosed that there was no spatial-arithmetic association in any delay condition (**Figure 2B**).

### Comparison between Horizontal and Vertical Spatial-Arithmetic Association

The results of the 2 × 2 × 2 × 3 ANOVA indicated that the main effect of delay was significant, *F*(2,52) = 42.372, *p* < 0.001, η^2^ = 0.620. In the 300 ms condition, the mean RT was faster than that in 150 ms condition, *F*(1,26) = 36.464, *p* < 0.001, η^2^ = 0.584, but not different from the 450 ms, *F*(1,26) = 0.944, *p* = 0.340, η^2^ = 0.035. We found a tendency of the interaction of dimension × operation × target side, although this effect was not significant, *F*(1,26) = 2.294, *p* = 0.192, η^2^ = 0.081.

In order to further quantify the strength of the interaction between operation and target side, Bayesian statistics for null-hypothesis significance testing (NHST) (Masson, 2011) were carried out for both horizontal and vertical dimensions. The result indicated that there was strong evidence for a horizontal spatial-arithmetic association (Bayes factor in favor of an effect = 16.23); in the vertical dimension, however, the strength of evidence tended to support the null hypothesis of no effect (Bayes factor in favor of null = 2.67) (Raftery, 1995).

### Influence of the Arithmetic RT on the Target Detection Task

Finally, given that the target detection task stimuli were triggered by oral responses to the proposal, it is possible that the RTs of the target detection task were affected by oral responses. We therefore checked the correlation between the RTs of these two tasks (**Figure 2C**). Statistical analysis (Lorch and Myers, 1990) revealed a small and non-significant correlation between the two variables (*r* = 0.033; *p* > 0.05).

## Discussion

In the current study, we adopted a target detection paradigm to investigate the spatial-arithmetic association in both horizontal and vertical dimensions within subjects. The results indicated that solving addition problems facilitated target detection on the right side, while targets on the left side were detected faster after solving subtraction problems. The finding confirmed that mental arithmetic induces spatial shifts of attention in horizontal space. However, we did not find this spatial-arithmetic association effect in vertical space. These two aspects of our results are now discussed in more detail.

Our results of a horizontal spatial-arithmetic association were consistent with a series of earlier studies (e.g., Masson and Pesenti, 2014; Mathieu et al., 2016; Liu et al., 2017; Masson et al., 2017). Some studies have proposed that the spatial attention shifts in mental arithmetic are induced by the spatial nature of number representation and processing (e.g., Masson and Pesenti, 2014; Mathieu et al., 2016). However, in our study the second operands and the proposed results were matched in addition and subtraction. Thus, the spatial biases in mental arithmetic were unlikely to be caused directly by the spatial representation of the specific numbers, but instead must be due to the arithmetic operation (Liu et al., 2017).

In elementary number processing, small magnitudes are typically associated with left/lower space and larger magnitudes with right/upper space (e.g., Schwarz and Keus, 2004; Holmes and Lourenco, 2012; Winter et al., 2015). In spatial arithmetic, a strong case has been made that subtraction is associated with left, and addition with right (e.g., Masson and Pesenti, 2014; Mathieu et al., 2016; Liu et al., 2017). This finding suggests a strong similarity between elementary number and arithmetic processing, and suggests that also subtraction could be associated with lower space and addition with upper space. However, we found no such vertical effect for mental arithmetic, although the Bayes factor analysis indicated that more data were needed to make this conclusive. Given that also in elementary number processing the horizontal bias is stronger than the vertical one (Holmes and Lourenco, 2012), more powerful designs are needed to determine whether there is a (small) vertical bias in mental arithmetic or none at all.

Thus, what is the nature of spatial shifts of attention underlying arithmetic operations? An earlier neural computational model proposed that elementary arithmetic is based on a cognitive system for spatial transformations that is “recycled” for the purpose of elementary arithmetic (Chen and Verguts, 2012). Consistent with this model, the neural structures recruited during addition and subtraction could resemble those observed during rightward or leftward eye movement, respectively (Knops et al., 2009a). The brain substrates implicated in spatial orienting even activate when an arithmetic operator (e.g., addition sign “+”) is shown (Mathieu et al., 2017). In this account, because arithmetic only hijacks the horizontal-dimension spatial transformation system, the spatial compatibility effect does not extend to the vertical dimension.

Other variables that may play an important role in the dissociated spatial-arithmetic associations in horizontal and vertical dimensions are the reading and writing direction (e.g., Dehaene et al., 1993), or subjects’ learning environment more generally. It has been suggested that left-to-right reading is responsible for the horizontal spatial-arithmetic association (Winter et al., 2015). However, for Chinese speakers (subjects used in the current study) the vertical reading direction is rarely used, which might thus cause the null effect in our vertical dimension. Furthermore, it is also plausible that the dissociated effects in our study might be caused by the fact that for Chinese speakers’ learning environment, mental arithmetic is performed from left to right but rarely from top to down. In contrast, elementary number processing typically occurs in both directions. Consistently, existing studies (see review, Winter et al., 2015), show that a horizontal direction is used in education for both elementary number processing and mental arithmetic; but that the vertical direction is used exclusively in education of elementary number processing. Future research should test the vertical spatial-arithmetic association in a Western (i.e., non-Chinese) population.

## Conclusion

In order to test the hypothesis that there is a similar system between elementary number processing and mental arithmetic we investigated the spatial-arithmetic associations in both horizontal and vertical dimensions. The dissociated effects in the current study cautions against overgeneralizations from elementary number processing to arithmetic based on number-space identity theories; and it points to the importance of considering individual subjects’ learning environment (Verguts and Chen, in press).

## Author Contributions

Conceived and supervised the experiments: QC and TV. Designed the experiments: QC and DL. Implemented the experiments and collected data: DL, ML, and ZL. Analyzed the results: DL. Wrote and revised the paper: DL, TV, ML, ZL, and QC.

## Conflict of Interest Statement

The authors declare that the research was conducted in the absence of any commercial or financial relationships that could be construed as a potential conflict of interest.

## Footnotes

1 http://www.gpower.hhu.de
